# A High-Efficiency Capacitor-Based Battery Equalizer for Electric Vehicles

**DOI:** 10.3390/s23115009

**Published:** 2023-05-23

**Authors:** Alfredo Alvarez-Diazcomas, Adyr A. Estévez-Bén, Juvenal Rodríguez-Reséndiz, Roberto V. Carrillo-Serrano, José M. Álvarez-Alvarado

**Affiliations:** 1 Facultad de Ingeniería, Universidad Autónoma de Querétaro, Las Campanas, Querétaro 76010, Mexico; aalvarez78@alumnos.uaq.mx (A.A.-D.); roberto.carrillo@uaq.mx (R.V.C.-S.); jmalvarez@uaq.edu.mx (J.M.Á.-A.); 2 Facultad de Química, Universidad Autónoma de Querétaro, Las Campanas, Querétaro 76010, Mexico; aestevez05@alumnos.uaq.mx

**Keywords:** electric vehicles, capacitor-based equalizers, battery management system, battery control

## Abstract

Technology in electric vehicles has increased substantially in the past decade. Moreover, it is projected to grow at record highs in the coming years since these vehicles are needed to reduce the contamination related to the transportation sector. One of the essential elements of an electric car is its battery, due to its cost. Batteries comprise parallel and series-connected cell arrangements to meet the power system requirements. Therefore, they require a cell equalizer circuit to preserve their safety and correct operation. These circuits keep a specific variable of all cells, such as the voltage, within a particular range. Within cell equalizers, capacitor-based ones are very common as they have many desirable characteristics of the ideal equalizer. In this work, an equalizer based on the switched-capacitor is proposed. A switch is added to this technology that allows the disconnection of the capacitor from the circuit. In this way, an equalization process can be achieved without excess transfers. Therefore, a more efficient and faster process can be completed. In addition, it allows another equalization variable to be used, such as the state of charge. This paper studies the operation, power design, and controller design of the converter. Moreover, the proposed equalizer was compared to other capacitor-based architectures. Finally, simulation results were presented to validate the theoretical analysis.

## 1. Introduction

Climate change has been pointed out as one of the significant challenges of humanity for the years to come. This climate change causes an increase in the average temperature worldwide. When greenhouse gases are emitted into the atmosphere, they create a thicker ozone layer. The ozone layer allows solar radiation to enter the atmosphere and retains part of this radiation. Therefore, by increasing the thickness of the ozone layer, the temperature of the planet rises. Many greenhouse gases emitted into the atmosphere are due to the transport sector. According to [1], around half of the oil produced in the world is consumed in transport, representing around one-quarter of greenhouse gas emissions. In addition, it is a sector with a high expectation of growth due to the increase in the quality of life worldwide [2].

Electric vehicles (EVs) emit practically zero greenhouse gases into the atmosphere during operation, so they have been presented as the eco-friendly alternative to vehicles powered by internal combustion engines. Therefore, many countries and regions have set goals for adopting EVs. For example, car manufacturers intend to sell 15 million electric cars annually from 2025 and onwards [3]. In addition, some countries have proposed their own goals, such as the case of India, which has proposed its roadmap so that by the year 2030, 40% of private vehicles and 100% of public transport will be electric [4]. In addition, the imbalance between cells is the main factor that reduces the useful life of the battery and decreases its total capacity with the time of use if a proper equalization is not carried out in the cells [5].

The safety and reliability of batteries used in electric vehicles pose significant challenges due to their unpredictable behavior in various operating conditions [6]. The Battery Management System (BMS) can provide the necessary actions when dangerous conditions are detected [7,8]. In this context, one approach to observing the behavior of a BMS is through a controlled environment simulation of battery performance [9].

Battery charging systems in electric vehicles are critical to safety and must address electrical hazards, overheating issues, electromagnetic compatibility, and battery life [10]. Designing proper charging systems and having battery monitoring systems in place is essential to ensure safe and efficient vehicle operation.

A series of policies have been implemented in various countries to stimulate the purchase of these vehicles to achieve the goals set. Some examples are credits for purchase, access to shared travel lanes, exemption from inspections, and reductions in registration fees, among others [11]. As an effect of these measures and the increase in social awareness, in recent years, great strides have been seen in the widespread adoption of EVs. The most significant example is China. In this country, the number of electric cars sold had a compounding annual growth rate of 48% from the year 2015 to the year 2018 [12]. In addition, the Chinese market accounts for 50% of the total sales in the world, followed by Europe and the United States in 2019 [13]. Due to the increase in the adoption of this type of vehicle and the goals set for the future, it is necessary to increase efforts to improve existing technologies to improve the user experience and stimulate EV sales.

In EVs, the battery is an essential element of the system. This element often represents the most expensive device, surpassing the chassis of the car. The batteries are made up of several cells connected in series and in parallel to meet the requirements of the traction system. However, the parameters of each cell are different, including internal resistance, capacity, self-discharge rate, etc. All cells do not behave equally. Therefore, after several charge and discharge cycles, each cell will have a different state of charge (SoC) [14,15].

When the cells of a battery bank do not present a similar SoC, this can cause overcharge or over-discharge in some cells. Moreover, these states cause the chemistry of the cells to be modified, and their useful life is reduced. Therefore, it is desirable to avoid these states in the cells. Preventing these behaviors with a chemical approach is difficult, since the parameter difference is due to a normal dispersion in the manufacturing process [16]. According to [17], there are five main factors that must be considered when deciding the monitoring technique to monitor the performance of a battery: accuracy, reliability, manufacturability, cost, and power.

**Figure 1:**
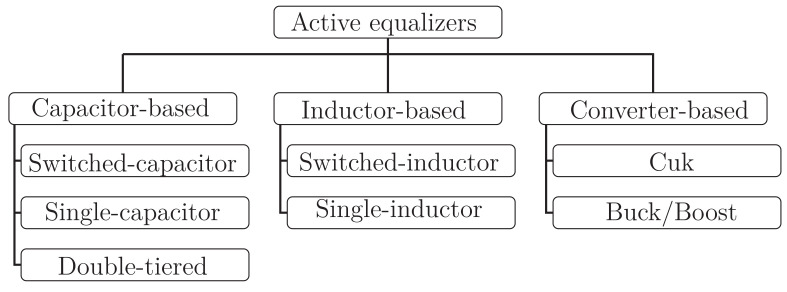
Classification of active equalizers considering the main element handling the transfers.

The best current solution is a circuit that takes active measures to maintain the SoC in all battery cells within an allowable range. The focus on this parameter is essential if the main purpose is to adequately manage the life and performance of batteries, because an unbalanced charge can decrease battery capacity and life [18]. This circuit is the battery equalizer. Battery equalizers not only increase the battery’s safety, but also improve the vehicle’s performance, since the battery can work in more extreme regions [19]. Figure 1 shows the main active equalizers proposed in the literature. This classification takes into account the main element used for energy transfers.

Within these schemes, one of the most interesting is represented by those based on capacitors. In one study [11], it was concluded that the switched-capacitor equalizer is the topology with the most desirable characteristics compared to an ideal equalizer. Among the desirable features, it exerts low stress on the components, does not need sensors, and does not require a complex controller. However, it has disadvantages, such as a long equalization time, many components, and poor efficiency.

**Figure 2:**
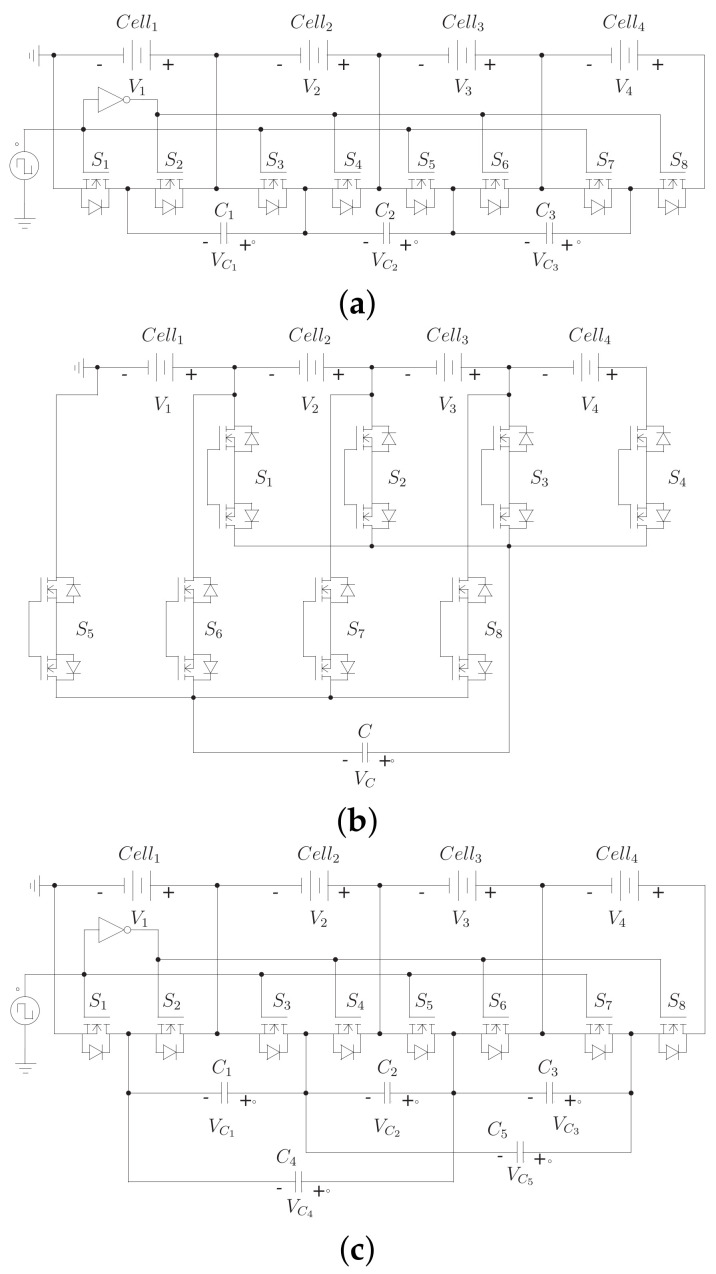
Classic architectures of BECs based on capacitors, (**a**) switched-capacitor topology, (**b**) single-capacitor topology, (**c**) double-tiered switched-capacitor topology.

Figure 2 shows this and other capacitor-based topologies. These topologies present the advantages already mentioned for the switched capacitor scheme. However, they have some peculiarities. The single-capacitor architecture offers a longer equalization time, since a single element has to handle all the transfers. However, this scheme allows different transfer possibilities, such as cell-to-cell, string-to-cell, string-to-string, and cell-to-string. However, a complex high-level controller is necessary to take advantage of these possibilities.

In addition, it features a low component count, although the capacitors are not the most expensive electronic elements. On the other hand, the topology of different layers decreases the equalization time, maintaining simple control. However, it increases the number of circuit elements and the stress on components, especially in the layers farthest from the battery bank.

Therefore, the switched-capacitor topology was taken as a base in this work, and some changes were made to obtain a more efficient scheme. Thus, the present manuscript is divided as follows. Section 2 presents the proposed topology; Section 3 gives the guidelines to design the circuit; in Section 4, the proposed converter is compared to other inductor-based equalizers, and Section 5 shows the simulation results. Finally, the main deductions of the investigation are presented in the conclusions.

## 2. Operation and Analysis of the Proposed Equalizer

In this section, the background of the work is presented in order to give a brief context of the model. Finally, the proposed equalizer for a four-cell battery bank is described.

### 2.1. Related Works

In the field of battery technology, it is crucial to address issues such as battery safety, electromagnetic compatibility, battery age, battery life cycle, and charging accuracy. Table 1 summarizes the main findings on EMS in these challenges. The presented state of the art shows great advantages in EMS techniques. However, the main challenge of the EMS in electric vehicles demands the higher performance of the equalization techniques to deal with the balance of the battery [20].

### 2.2. Contextualization of the Work

**Figure 3:**
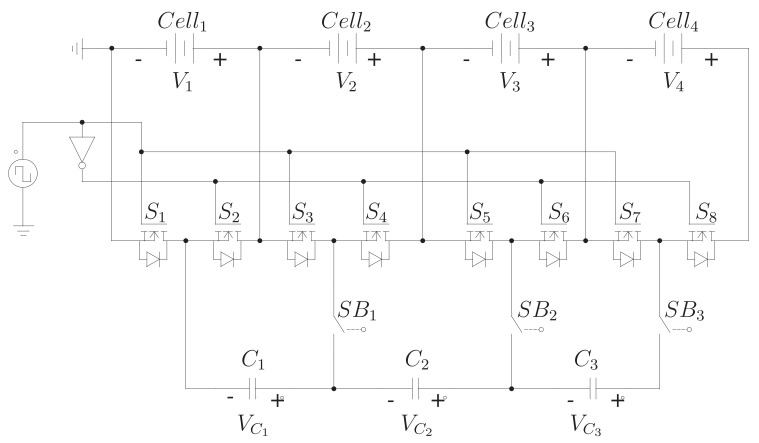
Proposed modified switched-capacitor equalizer.

Figure 3 shows the proposed equalizer for a four-cell battery bank. It can be noticed that it is similar to the switched-capacitor topology, but it adds a switch per capacitor to isolate this device from the circuit if needed. In one study [11], it is mentioned that one of the main disadvantages of this scheme is over-equalization. This undesired behavior is as follows: in the same equalization process, at one moment, one cell sends energy to another at first. However, before the process is complete, this energy transfer is reversed, and the cell that previously received now delivers energy. For instance, in the first stage, cell 1 provides energy to 2, and later, before the battery bank is equalized, cell 2 gives energy to cell 1. This behavior is undesirable because, in each transfer, there are power losses. Therefore, the efficiency of the equalization process decreases. The proposed architecture allows the capacitor to be disconnected from the circuit to avoid unwanted transfer and reconnected when the energy transfer is needed. However, it is necessary to introduce a controller to take advantage of this possibility.

In addition, the possibility of disconnecting the capacitor allows the use of another equalization variable other than the voltage. In the classical scheme, the capacitor voltage continuously oscillates between the voltage of the adjacent cells. Therefore, the voltage of the cells in the process is decisive. However, the voltage does not always reflect the internal state of the cell [15,31]. Thus, if another equalization variable, such as SoC, is used, a process ruled by this variable can be achieved. This feature is impossible with the classical switched-capacitor architecture.

**Figure 4:**
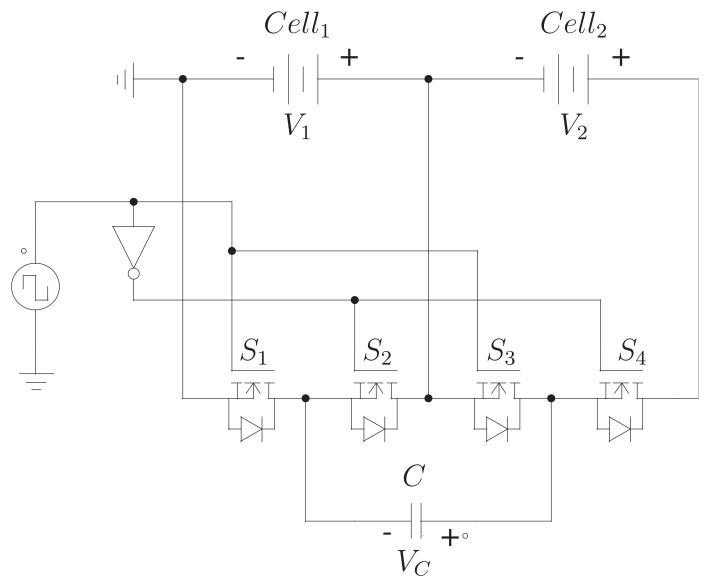
Module of the switched-capacitor scheme.

**Figure 5:**
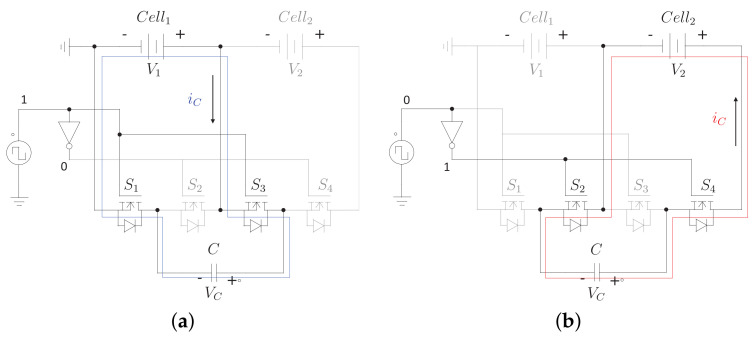
On and off state of the module of the switched-capacitor architecture, (**a**) on-state, (**b**) off-state.

Figure 4 shows the typical module of a switched capacitor equalizer. It can be seen that this module has two cells, four MOSFETs, and a capacitor. Additionally, Figure 5 shows the circuit in the on and off state of the switches. In this circuit, it can be seen how the capacitor alternates between being in parallel with one cell and then in parallel with the other. Therefore, the capacitor voltage oscillates between the voltages of both cells. When put in parallel with the cell with the highest voltage, it is charged until it reaches that same voltage. On the contrary, when forced in parallel with the cell with the lowest voltage, it delivers energy to the cell until both elements have the same voltage.

Equation (1) determines the behavior of the voltage across the capacitor in the on the state of the switches (Figure 5a). Where $v_{C}$ is the voltage across the capacitor, $I_{1}$ is the current through cell 1, and *C* is the value of the capacitance of the capacitor. Moreover, Equation (2) determines the behavior of the voltage across the capacitor in the off state of the switches (Figure 5b). In this equation, $v_{C}$ and *C* represent the same as in the previous equation. Moreover, $I_{2}$ is the current through cell 2.

(1) $$\frac{dv_{C}\left(t\right)}{dt}=\frac{I_{1}}{C}$$

(2) $$\frac{dv_{C}\left(t\right)}{dt}=-\frac{I_{2}}{C}$$

**Figure 6:**
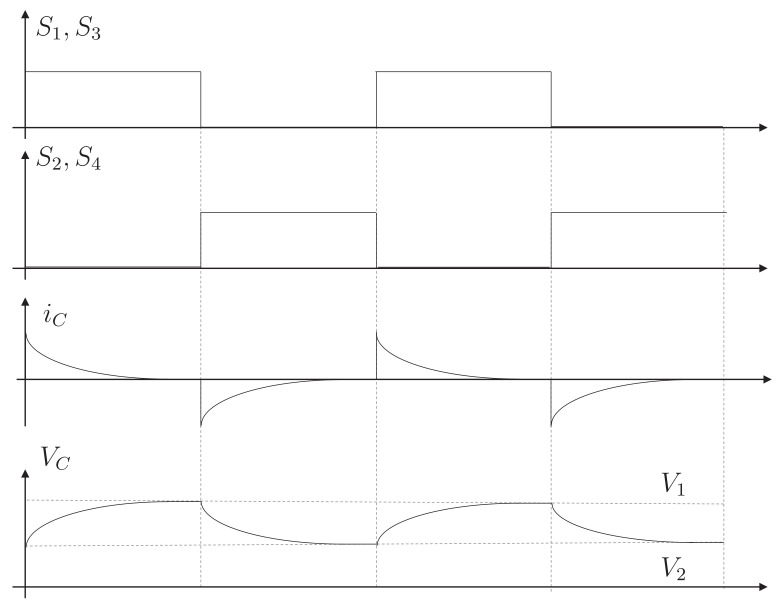
Waveform of the module of the switched-capacitor topology.

Figure 6 shows the behavior and steady-state waveforms of the module. In the first stage, the cell with a higher voltage ($V_{1}$) transfers energy to the capacitor by activating the proper switches ($S_{1},S_{3}$). Next, the correct combination of switches is induced ($S_{2},S_{4}$), and the power is transferred from the capacitor to the other cell ($V_{2}$). It can be seen that the voltage on the capacitor oscillates between the voltages of the cells to which it is connected. In addition, in the current graph, it can be seen that there is an initial peak, and then this variable behaves like a decreasing exponential. Equation (3) shows the equation that rules the behavior of the current in this type of circuit. Where $V_{1}$ is the voltage of cell 1, $V_{2}$ is the voltage of cell 2, $R_{eq}$ is the equivalent resistance of the current path, *t* is the time, and *C* is the capacitance of the capacitor.

(3) $$i_{C}\left(t\right)=\frac{V_{1}-V_{2}}{R_{eq}}e^{-\frac{t}{R_{eq}C}}$$

Equations (1)–(3) and Figure 6 show us the behavior of the circuit when the capacitors are connected to the circuit. However, in the proposed architecture, switches can isolate the capacitors from the circuit. If the switches are isolating the capacitors from the circuit, then there will not be transfers of energy between the related adjacent cells. This behavior represents the advantage provided by this topology. At work, ref. [11] over-equalization is mentioned as one of the main disadvantages of switched-capacitor equalizers. Over-equalization involves making more transfers than the minimum needed to equalize the battery bank. For example, consider the following case $V_{2}$ > $V_{1}$ > $V_{3}$. In this case, cell two will give energy to both adjacent cells in the first stage. However, there will come the point where $V_{1}$ = $V_{2}$ and $V_{3}$ will still be less than $V_{2}$. At this point, cell two will continue to send energy to cell three, and no more energy is transmitted from cell two to cell one. By continuing with the operation, cell two will have a lower voltage than cell one; therefore, cell one will transfer energy to cell two, and the direction of energy transfer will be reversed. This behavior is undesirable, since power losses are associated with every energy transfer. Therefore, power is being dissipated inefficiently in this topology. This new topology aims to avoid over-equalization by disconnecting the capacitor when the above situation can be predicted.

## 3. Design of the Proposed Equalizer

This section provides the guidelines for designing the proposed equalizer. The design process involves the power and control stages. A batter bank of four cells will be used as a use case, such as the one in Figure 3. However, it can be achieved with any number of cells.

### 3.1. Power Stage Design

The power stage design addresses the sizing of the MOSFETs, the bidirectional switch, and the capacitor. The necessary switches are sized to withstand the current and voltage of the application. Furthermore, another parameter to consider when picking one device is the desired switching speed for the application. Analyzing Figure 3, it can be seen that two MOSFETs are connected in parallel with one cell. Therefore, they must withstand half of the voltage in one cell. A typical 18,650 cell has a maximum voltage of 4.2 V. Thus, the maximum voltage for one MOSFET would be 2.05 V. Moreover, the maximum voltage for the capacitors is the maximum voltage in the cells (4.2 V) since they are forced in parallel. Finally, the maximum voltage required for the bidirectional switches is also the maximum voltage of the cells (4.2 V).

Equation (3) shows the behavior of the current in the capacitors. Therefore, Equation (4) shows the maximum current that can have all the elements in the path of the current: the cell, the bidirectional switch, the MOSFET, and the capacitor. Considering the typical series resistance of every component shown in Table 2, the maximum voltage of the cell is 4.2 V, and the minimum voltage of the cell is 2.5 V. The maximum current that must withstand the elements is 8.85 A.

(4) $$i_{C}\left(t\right)=\frac{V_{1}-V_{2}}{R_{eq}}$$

**Figure 7:**
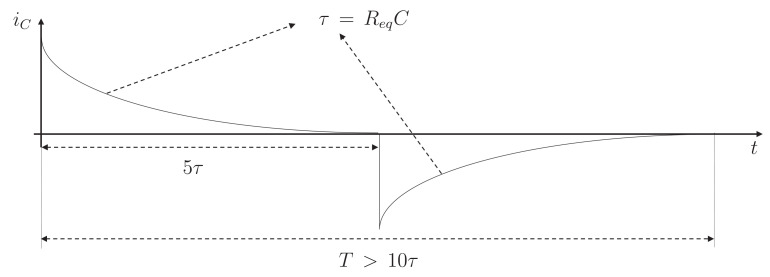
Typical current curve in the switched-capacitor equalizers.

In addition, it is necessary to select the switching frequency for the switches and the capacitance of the capacitor. Equation (3) shows that the time constant of the current is $R_{eq}C$. Furthermore, it is known that this type of system stabilizes at approximately five times the time constant. Therefore, the period of the square signal of the low-level controller must be greater than twice this time. This behavior is desirable because if the system has passed its settling time, the current will be zero, and a zero current switching converter is achieved. Therefore, the efficiency of the system is improved, since switching power losses can be neglected. Figure 7 shows a typical current curve for this circuit. Thus, you need a lower capacitance if you want a faster speed. This commitment must be taken into account in the design. Therefore, if a typical capacitance of 47 μF is selected, then a minimum period of 90.24 s and a maximum frequency of 11.08 kHz are required.

### 3.2. Controller Design

One of the significant advantages of the classic switched capacitor equalizer is its simple controller. The controller consists of a square wave signal generator. The value of this signal and the others are sent to the proper switches to achieve the transfers between adjacent cells. However, this simple controller leads to an undesirable over-equalization process. Therefore, a more complex controller will be required to take advantage of the disconnection allowed in the modified architecture proposed in this work.

**Figure 8:**
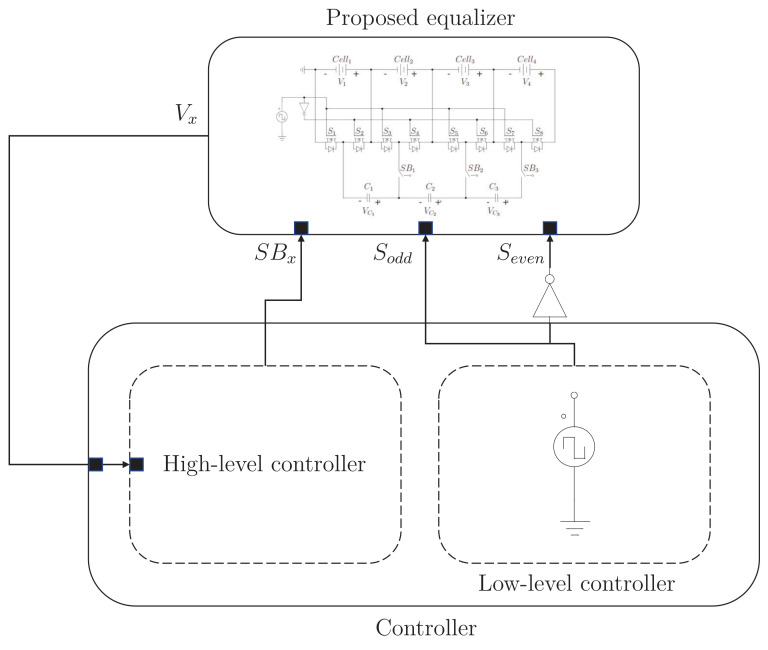
Architecture of the controller whit the proposed equalizer.

Figure 8 shows the architecture of the controller. It can be analyzed in two parts to make it easier to understand. A straightforward controller consisting of a square wave signal generator is needed at a low level. This controller is the same one used in the classical switched-capacitor equalizer. In addition, there is a high-level controller which helps us decide when to connect/disconnect the capacitors of the circuit to avoid over-equalization. The controller necessitates measuring the voltage in every cell and acting on the switches that isolate the capacitors from the rest of the circuit to disconnect the proper capacitor. The switches introduced in the proposed equalizer ($SB_{1}$, $SB_{2}$, and $SB_{3}$ of Figure 3) are the ones that allow the capacitors to be disconnected from the circuit and, thus, avoid over-equalization.

**Figure 9:**
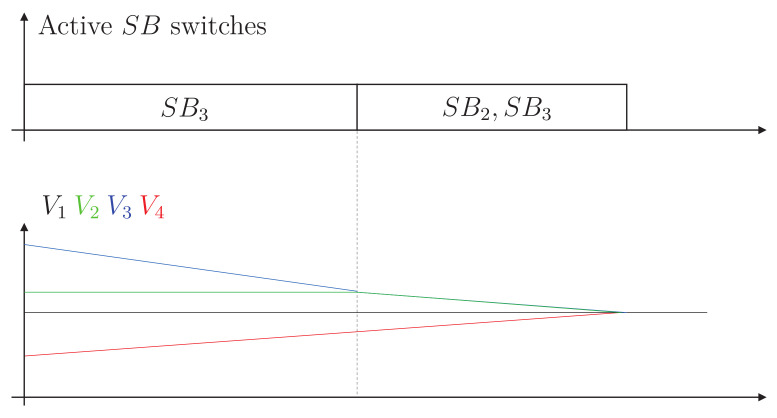
Operation of the proposed topology.

Figure 9 shows the operation of the converter in a use case that takes advantage of the possibility of disconnection of the capacitors. The idea of the controller is to predict over-equalization. This prediction is made by comparing the voltage in each cell with the average voltage of the cells in the battery bank. The average voltage is the reference point, since the voltage in all cells should tend to this value at the end of the equalization process. In this figure, it is necessary to assume that $V_{1}$ equals the average voltage between the four cells. Therefore, all the cells will have that voltage at the end of the equalization process, and $V_{1}$ is not considered for this analysis, since it already achieves the target voltage for the equalization process. The figure shows a first stage in which the voltage $V_{3}$ exceeds the voltage of adjacent cells $V_{2}$ and $V_{4}$. In the classical topology, cell three would send energy to both cells. However, the controller must predict that the voltage in cell 2 is higher than the average of the bank. Therefore, it must give up energy at some point, and receiving it does not make sense. Thus, the figure shows the first stage in which the capacitor that manages the energy transfers between cells 2 and 3 is disconnected. This behavior is maintained until the controller recognizes that it must connect that capacitor. At this point, the second stage begins with capacitors $C_{2}$ and $C_{3}$ connected until battery bank equalization is achieved without cell over-equalization.

**Figure 10:**
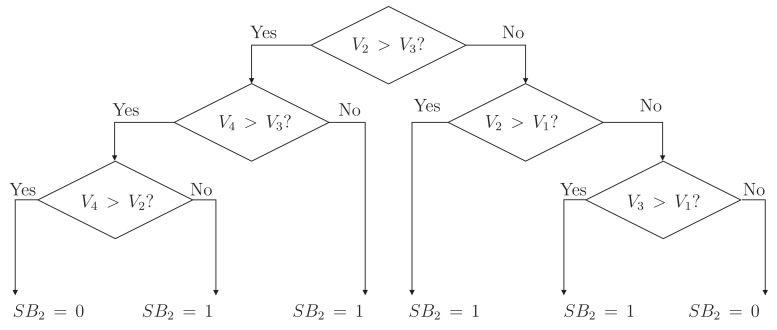
Flowchart diagram of the controller for $SB_{2}$ switch.

Figure 10 represents the flowchart diagram of the high-level controller for switch $SB_{2}$. However, the analysis for this switch can be extended to the rest of the $SB_{x}$ switches. It can be seen how the voltage between adjacent cells is considered to make the decision. If, for example, $V_{2}$>$V_{3}$ and $V_{3}$ > $V_{4}$, the decision to turn on the switch is evident since the energy must go in the direction from cell two to four. However, it is unclear when the voltage $V_{3}$ is less than $V_{2}$ and $V_{4}$ since over-equalization can occur and reverse transfers. In this case, the voltage of cells two and four must be compared to decide the state of switch $SB_{2}$.

## 4. Comparison

In this section, the architectures of Figure 3 are compared with the proposed topology to highlight their advantages and disadvantages. The parameters to consider are the number of elements, stress on components, equalization time, controller complexity, and efficiency.

Table 3 summarizes the number of components for each topology. It can be seen that the proposed architecture has the same number of elements as the switched capacitor, but a relay is added per capacitor. Additionally, the single-capacitor equalizer only has one capacitor but many MOSFETs. These devices are expensive and require a complex high-side driver to operate. Finally, the double-tiered capacitor scheme has the same number of elements as the switched-capacitor, plus a second layer of capacitors is added. Adding more capacitors is often undesirable because they are less reliable devices with a shorter lifespan than other electronic elements.

Analyzing the switched-capacitor equalizer, it can be seen that it forces each capacitor in parallel with the cells. Therefore, the capacitors must withstand the voltage of each cell (maximum of 4.5 V). On the other hand, the double-tiered switched-capacitor must support the cell voltage for the first row of capacitors. However, the second row must support the sum of two cells, and so on, if it has more rows. The single-capacitor topology depends on the controller. This scheme is very flexible and allows any transfer. Therefore, if the controller only considers cell-to-cell transfers, then it must support the voltage of one cell. However, if the controller can use pack-to-cell transfers, it must withstand the voltage of the entire battery bank. In this case, the greater the stress on the components it must support, the shorter the equalization time. Finally, the proposed architecture must withstand the voltage of one cell, since it is similar to the switched-capacitor scheme.

As Equation (4) shows, the maximum current will be directly proportional to the difference in voltage between the cells and inversely proportional to the resistance in the path of the current. In the double-tiered switched-capacitor topology, the current in the second row of capacitors tends to be higher than in the first row because there are two cells, so the difference between their voltages will be higher. On the other hand, the single-capacitor architecture, as in the voltage, depends on its controller. If it only considers cell-to-cell transfers, it will have the same stress as the switched-capacitor equalizer. However, if it considers pack-to-cell transfers, it will have a greater difference between the cell voltages and must withstand a higher current. Finally, the proposed converter must support a current slightly lesser than the switched capacitor’s. When introducing the relay as an additional element to the current path, it will have a slightly higher equivalent resistance.

The equalization time is closely related to the number of elements that transfer energy. In this case, the single-capacitor equalizer will take the longest, since it only uses a component to perform all the necessary transfers. At the same time, the fastest will be the double-tiered switched-capacitor. The proposed equalizer should be slower than the classic switched-capacitor since it has the same number of elements. However, in the proposed controller it is disconnected from the capacitors at times. Therefore, it should take longer to achieve bank equalization. On the other hand, the controller complexity is the same for both the switched-capacitor and the double-tiered switched-capacitor. These topologies only require a square signal on the switches. In the case of the single-inductor scheme, you need a high-level controller to decide which cell or string of cells will be the source and which will be the destination. Finally, the proposed scheme requires a high-level controller to determine when to disconnect each capacitor.

**Figure 11:**
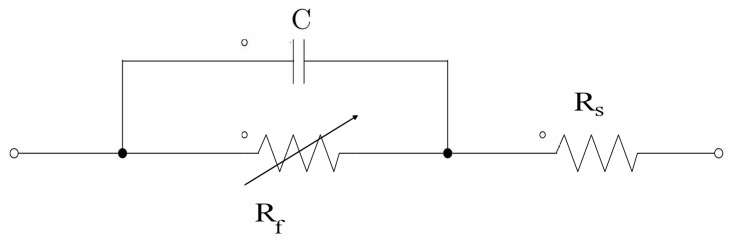
Faradaic leakage resistance in the proposed circuit.

Power losses are another critical parameter to compare the proposed equalizer. Equation (5) shows the conduction losses in the switched-capacitor scheme, where $i_{C}$ is the current in the capacitor, *D* is the duty cycle of the square wave signal, $R_{on\_B_{x}}$ is the series resistance of the cell *x*, $R_{on\_S_{x}}$ is the series resistance of the MOSFET *x*, $R_{C}$ is the series resistance of the capacitor. Finally, for the development of the Proposed modified switched-capacitor equalizer, the Faradaic leakage resistance was considered in parallel with the capacitor [35]. Figure 11 displays the connection of the Resistance in the circuit [36].

If it is assumed that the resistance of the cells and the MOSFETs have the same value, and the duty cycle is 50%, then the Equation (6) is obtained.

(5) $$\begin{array}{cc} P_{Cond} & =i_{C}^{2}(D\left(R_{on\_B_{1}}+R_{on\_S_{1}}+R_{on\_S_{3}}+R_{C}\right)\\ \; & +\left(1-D\right)\left(R_{on\_B_{2}}+R_{on\_S_{2}}+R_{on\_S_{4}}+R_{C}\right) \end{array}$$

(6) $$P_{L\_Cond}=i_{L}^{2}\left(R_{on\_B}+2R_{on\_S}+R_{C}\right)$$

On the other hand, Equation (7) shows the conduction losses in a transfer for the proposed equalizer, shere $R_{on\_SB}$ represents the on-resistance of a switch SB. If we consider the values of the Table 2, the typical resistance in the switched-capacitor architecture is 164 mΩ. In comparison, the proposed equalizer has a resistance of 224 mΩ. Therefore, for a transfer, the classic equalizer has a power loss representing 73.2% of the proposed one.

(7) $$P_{L\_Cond}=i_{L}^{2}\left(R_{on\_B}+2R_{on\_S}+R_{on\_SB}+R_{C}\right)$$

## 5. Simulation Results

According to [9], for a BMS, there are two types of tests: the functional test, which focuses on verifying that the BMS system can perform its functions correctly, and the non-functional test, which focuses on verifying how the components system work together to meet system requirements. Simulation can be a useful tool to carry out non-functional tests because this provides a controlled environment for testing scenarios and conditions that could be dangerous or difficult to test in real life, such as battery fault detection and protection, interaction with other systems, and communication capabilities before they are employed in a real scenario [37,38]. In this work, simulations were carried out with the PSIM software developed for power electronics. Table 4 shows the values used in the elements of the simulation.

**Figure 12:**
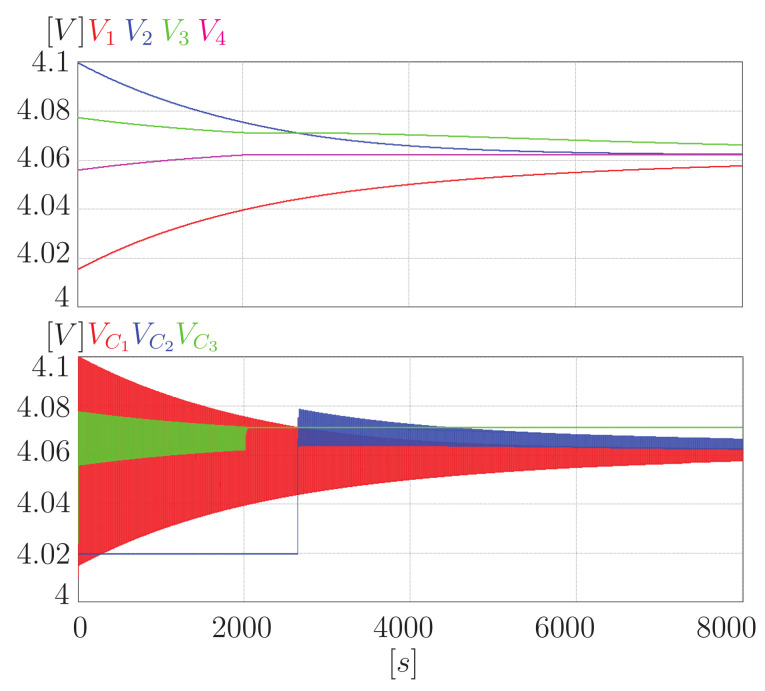
Equalization of a four-cell battery bank using the proposed equalizer.

Figure 12 shows the equalization process for a four-cell battery bank using the proposed equalizer. It can be seen that the voltage in all the cells of the bank tends to a common point, achieving the equalization of the battery. In addition, the expected behavior in the voltage of the capacitors can be appreciated. This voltage oscillates between the voltage of the adjacent cells that equalize. However, it can be seen that $V_{C_{3}}$ reaches a point where it no longer oscillates. This behavior is because the capacitor was disconnected from the circuit to prevent over-equalization. Similar behavior can be seen in $V_{C_{2}}$, only this capacitor is initially unconnected and is then connected to the circuit.

**Figure 13:**
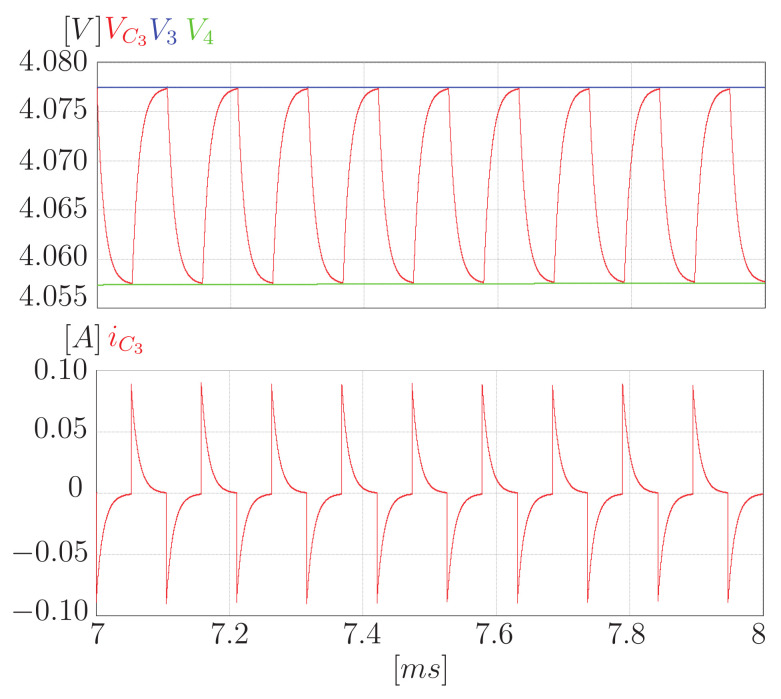
Zoom of the current and the voltage on capacitor $C_{3}$.

In addition, Figure 13 shows a zoom to the variables of interest in the proposed converter. You can see the expected behavior. The voltage of the capacitor oscillates between the voltage of the cells. Moreover, current peaks then fall to zero as a decreasing exponential. It is important to note that the change in the state of the switches with zero current causes no switching losses.

**Figure 14:**
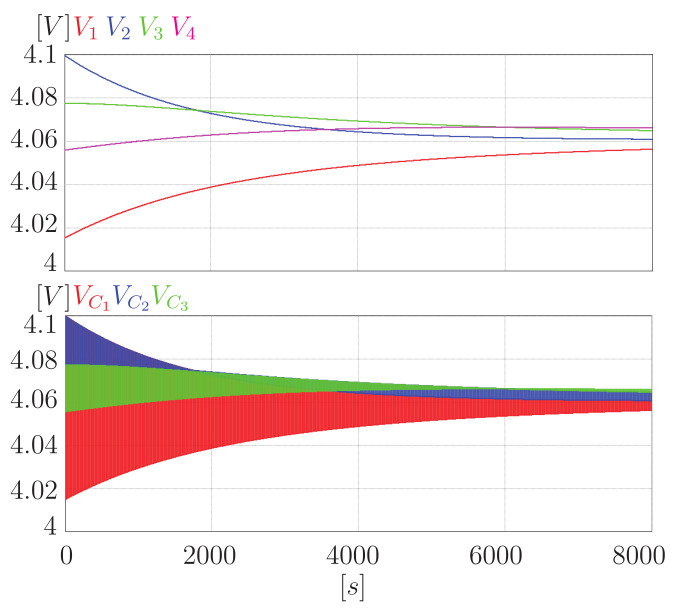
Equalization of a four-cell battery bank using the switched-capacitor equalizer.

Figure 14 shows a simulation of the equalization process with the same conditions using the classic switched-capacitor equalizer. When compared with the previous figure, you can see the difference in the behavior of the voltage of the capacitors. In this case, they are always connected and oscillating between the voltage of the cells. This behavior leads to over-equalization and is precisely what the proposed equalizer avoids.

Considering a switching frequency of 9.5 kHz, in 8000 s, the square signal has 152 million changes. In addition, it is necessary to consider that each change is a transfer. Therefore, if you have three capacitors, 456 million transfers were made. On the other hand, a counter was placed in each switch to determine how many transfers were made in the proposed equalizer, resulting in 298,720,000 transfers. Therefore, equalization is achieved, requiring approximately 65.5% of the transfers.

Furthermore, it is necessary to remember that in the classic equalizer, the power lost in a transfer represents 73.2% of the power lost in a transfer of the proposed equalizer. Therefore, the power lost in the equalization process in the proposed equalizer is 89.47% of the energy lost in the classic equalizer. Finally, when the process finished in 8000 s, the difference between the cell with the highest voltage and the one with the lowest voltage was 10 mV for the switched-capacitor scheme and 9 mV for the proposed equalizer. Therefore, avoiding over-equalization also impacted the equalization time. This behavior makes sense since if only the necessary transfers are made, the equalization time should be less.

## 6. Conclusions

This work presented the analysis and design process of the proposed circuit. Moreover, the basic module of the switched-capacitor scheme was analyzed. The design of the controller was shown. It was divided into two subsystems: a low-level controller consisting of a square-wave signal and a high-level controller ruling the connection/disconnection of the capacitors of the circuit. Simulations to validate the theoretical assumptions were presented. These simulations confirmed the expected behavior in the variables of the circuit.

This work showed a novel modified switched-capacitor equalizer for its application in EVs. The main difference in the proposed architecture is that it allows the disconnection of the capacitors from the circuit. This possibility can be used to avoid over-equalization, which has been pointed out as one of the main problems with this circuit. The proposed architecture achieved higher efficiency and a decreased equalization time. Finally, it was compared with other capacitor-based equalizers present in the literature. The parameters considered were the component count, stress on components, amount of sensors required, process speed, and controller complexity. Additionally, the capacitor disconnection of the circuit can be used to equalize another variable besides voltage, such as the SoC.

## Figures and Tables

**Table 1:** Related works of EMS in batteries for electric vehicles.

Author	Year	Model	Experiments	Results
[21]	2018	Optimization of electrochemical model-based moving horizon estimation (MHE)	Comparison with extended Kalman filtering (EKF) and unscented Kalman filtering (UKF) in terms of accuracy and fault tolerance	MHE is more accurate than the EKF and UKF models but problematic when implemented.
[22]	2018	Extended Kalman Filter-based online parameter identification with a smooth variable structure filter.	Validation by numerical simulation and experimental results in MATLAB.	Applicable to any type of battery with an SOC error of about 2%
[23]	2018	Linear aging model for remaining useful life and part load voltage curve.	Battery capacity estimation with low computational cost and predict RUL offline.	Estimation errors of up to 1.5%.
[24]	2018	Use of Advanced Vehicle Simulator for modeling.	Performance of the power system of the Fuel-Cell Hybrid Electric Vehicle in different aspects.	Battery SOC changes by less than 2%.
[25]	2019	Estimation of pulse power performance by considering the pulse duration of Lithium Battery and Supercapacitor.	Analyzing the equivalent model of a battery and the supercapacitor with series resistance and more than two RC ladders.	For a lithium battery module, a 44.4 V, 11 Ah, and a supercapacitor with 36 V, 30 F was presented.
[26]	2021	EMS based on deep reinforcement learning.	After the training process, the effectiveness of the trained EMS is validated under four new driving profiles.	The main energy source, as its SoC decreases from 90% to 67% during the validation process.
[27]	2021	Cloud-based multi-objective energy management strategy.	Controls the power split between two battery packs.	The model achieves 50.8 kJ and 49.5 kJ less energy loss.
[28]	2021	Estimating SoC by advanced hysteresis modeling method.	Use of Kalman filter and autoregressive exogenous to estimate the parameters of a LiFePO${}_{4}$.	The model achieved around root mean square error of 0.69% and a mean absolute error of 0.47%.
[20]	2021	Bipolar-resonant LC converter for a multicell-to-multicell equalizer.	The proposed method was compared against five conventional equalization models for n cells/modules.	The proposed equalizer achieved 84.84% to 91.68% of efficiency when balancing powers from 1.426 W to 12.559 W in 8 lithium-ion battery cells
[29]	2022	The authors use a simulation model of a hybrid electric vehicle that includes a fuel cell, battery, and supercapacitor and evaluate the vehicle’s performance using a standard drive cycle.	A Matlab simulation model of a hybrid electric vehicle that includes a fuel cell, a battery, and a supercapacitor was carried out	The proposed hybrid strategy also reduces the energy demand of the vehicle’s heating and cooling system, helping to reduce energy consumption and improve the vehicle’s energy efficiency.
[30]	2023	Energy management strategy based on reinforcement learning using the Double Deterministic Reinforcement Learning (TD3) algorithm with a non-parametric reward function.	The experiments were carried out in an electric vehicle simulator	The results showed that the proposed energy management strategy achieved a reduction in energy consumption of 7.6% and an increase in driver satisfaction of 3.3% compared to a conventional energy management strategy.

**Table 2:** Typical resistance of all the components used in the proposed equalizer.

Component	Typical Resistance [mΩ]
Cell	50
MOSFET	32
Bidirectional switch	60
Capacitor	50

**Table 3:** Component count for each capacitor-based equalizer considering *n* cells.

Equalizer	*n* Cells
Switched-capacitor [32]	n−1 capacitors 2n MOSFETs
Double-tiered switched-capacitor [33]	2n−3 capacitors 2n MOSFETs
Single-capacitor [34]	1 capacitor 4n MOSFETs
Proposed equalizer	n−1 capacitors 2n MOSFETs n−1 SPDTs

**Table 4:** Value of the components used in the simulation.

Component	Value
Battery cell	*Q* = 2500 mAh, $I_{max}$ = 4 A
Initial voltages	$V_{1}=4.01\;V$, $V_{2}=4.1\;V$ $V_{3}=4.08\;V$, $V_{4}=4.06\;V$
Capacitor	47 μF
Switching frequency	9500 Hz

## Data Availability

Not applicable.
